# Motor and Sensory Features of Cervical Dystonia Subtypes: Data From the Italian Dystonia Registry

**DOI:** 10.3389/fneur.2020.00906

**Published:** 2020-08-26

**Authors:** Francesca Di Biasio, Roberta Marchese, Giovanni Abbruzzese, Ottavia Baldi, Marcello Esposito, Francesco Silvestre, Girolamo Tescione, Alfredo Berardelli, Giovanni Fabbrini, Gina Ferrazzano, Roberta Pellicciari, Roberto Eleopra, Grazia Devigili, Francesco Bono, Domenico Santangelo, Laura Bertolasi, Maria Concetta Altavista, Vincenzo Moschella, Paolo Barone, Roberto Erro, Alberto Albanese, Cesa Scaglione, Rocco Liguori, Maria Sofia Cotelli, Giovanni Cossu, Roberto Ceravolo, Mario Coletti Moja, Maurizio Zibetti, Antonio Pisani, Martina Petracca, Michele Tinazzi, Luca Maderna, Paolo Girlanda, Luca Magistrelli, Salvatore Misceo, Marcello Romano, Brigida Minafra, Nicola Modugno, Marco Aguggia, Daniela Cassano, Giovanni Defazio, Laura Avanzino

**Affiliations:** ^1^IRCCS Policlinico San Martino, Genoa, Italy; ^2^Department of Neuroscience, Rehabilitation, Ophtalmology, Genetics and Maternal Child Health, University of Genoa, Genoa, Italy; ^3^Department of Neurosciences, Reproductive Sciences and Odontostomatology, Federico II University of Naples, Naples, Italy; ^4^“Salvatore Maugeri” Foundation, Institute of Telese Terme (BN), Benevento, Italy; ^5^Department of Human Neurosciences, Sapienza University of Rome, Rome, Italy; ^6^IRCSS Neuromed, Pozzilli, Italy; ^7^Department of Basic Science, Neuroscience and Sense Organs, Aldo Moro University of Bari, Bari, Italy; ^8^Fondazione I.R.C.C.S. Istituto Neurologico Carlo Besta, UOC Neurologia 1, Milan, Italy; ^9^Neurology Unit, Center for Botulinum Toxin Therapy, A.O.U. Mater Domini, Catanzaro, Italy; ^10^Neurologic Unit, University Hospital, Verona, Italy; ^11^San Filippo Neri Hospital, ASL Roma 1, Rome, Italy; ^12^Department of Medicine, Surgery and Dentistry “Scuola Medica Salernitana”, Neuroscience Section, Universitá di Salerno, Baronissi, Italy; ^13^IRCCS Humanitas Research Hospital Humanitas, Milan, Italy; ^14^IRCCS Institute of Neurological Sciences, Bologna, Italy; ^15^Neurology Unit, ASST Valcamonica, Esine, Italy; ^16^Neurology Service and Stroke Unit, Department of Neuroscience, AO Brotzu, Cagliari, Italy; ^17^Neurology Unit, Department of Clinical and Experimental Medicine, University of Pisa, Pisa, Italy; ^18^Neurology Unit, Umberto I Hospital, Turin, Italy; ^19^Department of Neuroscience 'Rita Levi Montalcini', University of Turin, Turin, Italy; ^20^Neurology, Department of Systems Medicine, University of Rome Tor Vergata, Rome, Italy; ^21^Fondazione Policlinico Universitario A. Gemelli – IRCCS, Rome, Italy; ^22^Institute of Neurology, Università Cattolica del Sacro Cuore, Rome, Italy; ^23^Department of Neuroscience, Biomedicine and Movement, University of Verona, Verona, Italy; ^24^Department of Neurology and Laboratory of Neuroscience, IRCCS Istituto Auxologico Italiano, Milan, Italy; ^25^Department of Clinical and Experimental Medicine, University of Messina, Messina, Italy; ^26^Movement Disorders Centre, Neurology Unit, Department of Translational Medicine, University of Piemonte Orientale, Novara, Italy; ^27^PhD Program in Clinical and Experimental Medicine and Medical Humanities, University of Insubria, Varese, Italy; ^28^Neurologic Unit, San Paolo Hospital, Bari, Italy; ^29^Neurology Unit AOOR Villa Sofia Cervello, Palermo, Italy; ^30^Parkinson's Disease and Movement Disorders Unit, IRCCS Mondino Foundation, Pavia, Italy; ^31^Neurology Department, Asti Hospital, Asti, Italy; ^32^Unit of Neurology, Ospedale Maria Vittoria, Turin, Italy; ^33^Neurology Unit, Department of Medical Science and Public Health, University of Cagliari, Cagliari, Italy; ^34^Department of Experimental Medicine, Section of Human Physiology, University of Genoa, Genoa, Italy

**Keywords:** cervical dystonia, tremor, sensory trick, pain, spread

## Abstract

**Introduction:** Cervical dystonia (CD) is one of the most common forms of adult-onset isolated dystonia. Recently, CD has been classified according to the site of onset and spread, in different clinical subgroups, that may represent different clinical entities or pathophysiologic subtypes. In order to support this hypothesis, in this study we have evaluated whether different subgroups of CD, that clinically differ for site of onset and spread, also imply different sensorimotor features.

**Methods:** Clinical and demographic data from 842 patients with CD from the Italian Dystonia Registry were examined. Motor features (head tremor and tremor elsewhere) and sensory features (sensory trick and neck pain) were investigated. We analyzed possible associations between motor and sensory features in CD subgroups [focal neck onset, no spread (FNO-NS); focal neck onset, segmental spread (FNO-SS); focal onset elsewhere with segmental spread to neck (FOE-SS); segmental neck involvement without spread (SNI)].

**Results:** In FNO-NS, FOE-SS, and SNI subgroups, head tremor was associated with the presence of tremor elsewhere. Sensory trick was associated with pain in patients with FNO-NS and with head tremor in patients with FNO-SS.

**Conclusion:** The frequent association between head tremor and tremor elsewhere may suggest a common pathophysiological mechanism. Two mechanisms may be hypothesized for sensory trick: a gating mechanism attempting to reduce pain and a sensorimotor mechanism attempting to control tremor.

## Introduction

Cervical dystonia (CD) is one of the most common forms of adult-onset idiopathic isolated dystonia and is characterized by involuntary muscle contractions, causing abnormal twisted postures of the head and neck (1, 2).

Little is known about the pathophysiology of CD: an abnormal neural processing in basal ganglia-motor cortical network and in the cerebellum is now considered the main mechanism underlying this disorder (2–4). One hypothesis is that the key nodes in the malfunctioning cerebral network may distinguish between different CD phenotypes (5, 6). Indeed, one feature of CD is the heterogeneity and variability of the clinical signs of these patients.

So far, it has been suggested that the presence of tremor in CD may indicate a more widespread and complex pathological cerebral network, likely encompassing a pivotal role of the cerebellum (7). As a matter of fact, tremor in CD is associated with changes in sensory processing and motor adaptation tasks that are heavily modulated by cerebellar processing, including eyeblink classical conditioning (8), anticipatory adjustment during motor adaptation (9), proprioceptive acuity assessment (10), and human motion perception (11).

Following the line of reasoning that directed the identification of tremulous phenotype in dystonia as a distinct pathophysiological phenotype, a further step might be accomplished by exploring whether other aspects of the dystonia clinical spectrum may hide a distinctive pathophysiological basis.

Even if it has not been supported so far by a strong neurobiological background, contemporary classification of CD has also divided it into subtypes specific to the site of onset and spread of dystonia (12). Precisely, Norris et al. recently proposed a clinical classification of CD, according to the site of onset and spread of dystonia and described different subgroups, that may correspond to a distinctive clinical entities with different pathophysiological mechanisms (12). They classified the patients in four different subtypes: (i) focal neck onset with no spread to adjacent body segment (FNO-NS); (ii) focal neck onset with segmental spread (FNO-SS); (iii) focal onset elsewhere with segmental spread to neck (FOE-SS); (iv) segmental neck involvement without spread (SNI).

Examining possible differences between prevalence of tremor and sensory trick in the various subtypes, they showed that tremor of the dystonic regions was more frequent in patients with focal neck onset with segmental spread than in patients with focal onset elsewhere and later spread to the neck. This finding supports prior reports that dystonia more frequently spreads in patients with tremor compared to those without (13). They also found that sensory tricks were less frequent in patients with focal onset elsewhere and later spread to neck subgroups compared with patients with focal neck onset with segmental spread and focal neck onset with no spread, suggesting that the presence of a sensory trick in CD is related to the site of onset in the neck rather than dystonia distribution or dystonia spread (12).

Based on the above-mentioned findings, a further development in the definition of the pathophysiological basis of these CD subtypes may be to investigate whether motor and sensory features, that are additional to dystonic contractions and that *per se* differentiate CD subtypes, could also cluster together in the CD subtypes. In order to obtain this information, a registry-based dataset is the ideal tool for the data collection of large-sized sample with harmonized data collection protocols, even if hampered by the unavailability of objective data. Taking advantage of the Italian Dystonia Registry (IDR) database, we investigated here, for the first time in a large cohort of CD patients, the relationship between motor (head tremor and tremor elsewhere) and sensory (sensory trick and pain) features in the various CD subtypes, as classified by Norris et al. on the basis of the spreading of dystonia (12). We expect to depict different scenarios of sensorimotor associations in CD subtypes, based on the site of onset of dystonic symptoms and the presence of spread, suggesting different pathophysiological mechanisms underpinning variability of clinical course and manifestation.

## Materials and Methods

Data were obtained from the Italian Dystonia Registry (IDR) database (14). Thirty-seven Italian institutions contribute to the database using a common clinical protocol. Eligibility for the IDR requires a diagnosis of dystonia according to published criteria (1, 15) and age at dystonia onset >17 years (14). We excluded patients with CD secondary to known causes such as medication-induced dystonia and parkinsonian syndromes, and those with orthopedic procedures that may have affected neck movement.

Patients' assessment includes standardized historical data collection and clinical examination (14). In order to examine the clinical course of dystonia the year of dystonia onset was recorded for each affected body region and the spread from the referred clinical onset was also addressed. Data from the first visit were only analyzed if a participant had additional follow-up visits. No patient was excluded because of insufficient/missing data. Tremor was classified in tremor affecting different body segments: (i) head; (ii) upper limbs; (iii) lower limbs; (iv) other segments. Data on sensory trick were collected based on a “yes/no” answers/output.

We divided the patients according to the classification proposed by Norris et al. (12): (i) FNO-NS, (ii) FNO-SS, (iii) FOE-SS; (iv) SNI. We analyzed the following data: (i) demographic features (age, sex); (ii) disease duration; (iii) presence of head tremor and tremor elsewhere (yes/no); (iv) presence of sensory trick (yes/no); (v) presence of neck pain (yes/no). All patients gave informed consent to participate in the study, which was approved by the local ethical committee of each institution.

### Statistical Analysis

Statistical analysis was performed with IBM SPSS version 23. Data are presented as mean ± standard deviation for continuous variables and as percentage for categorical variables. Differences between groups for the variables analyzed were tested with unpaired *t*-test and Chi square test. The associations between motor (head tremor and tremor elsewhere) and sensory (sensory trick and pain) features in CD subtypes were tested by means of Chi-square test. Then, a logistic regression analysis was done on significant associations, adding as covariates disease duration, sex and age. For all analysis, significance was set at the 0.05 level.

## Results

Data from 842 patients with cervical dystonia were analyzed (Table 1). The majority were female (62.9%). The mean disease duration was 14 years. Neck pain was present in 55.7% of patients. Head tremor was present in 47.98% and tremor elsewhere was present in 11.75% of CD. The sensory trick was present in 30.71% of patients. When CD patients were classified according to onset site of dystonia and spread, 70% of participants were classified as FNO-NS, 10% as SNI, 8% as FNO-SS and 12% as FOE-SS.

**Table 1:** Demographic data.

		**No Spread**		**Spread**	
	**All**	**FNO-NS**	**SNI**	**FNO-SS**	**FOE-SS**
Number of subjects	842	594 (70%)	85 (10%)	66 (8%)	97 (12%)
Woman	527 (62.9%)	373 (62.7%)	57 (67.1%)	37 (56%)	60 (61.9%)
Mean Age ± SD	60.7 ± 14.5	60.2 ± 13.9	58.8 ± 15.7	63.1 ± 15.7	63.5 ± 15.7
Mean Disease duration ± SD	14 ± 13.5	13.8 ± 11.1*^°^^§^^#^*	19.9 ± 18.1	19.7 ± 14.5	19.1 ± 14.6
Sensory trick	259 (30.7%)	182 (30.6%)	19 (22.4%)	24 (36.4%)	35(36.1%)
Pain	469 (55.7%)	331 (55.6%)	48 (56.5%)	35 (53%)	55 (56.7%)
Head tremor	404 (47.9%)	286 (48.1%)	36 (42.4%)	32 (48.5%)	50 (51.5%)
Tremor elsewhere	99 (11.8%)	32 (5.4%)*^°^^§^^#^*	22 (25.9%)	21 (31.8%)	24 (24.7%)

FNO-NS, focal neck onset, no spread; SNI, segmental neck involvement; FNO-SS, focal neck onset, segmental spread; FOE-SS, focal onset elsewhere, segmental spread to neck; SD, standard deviation. Statistical significant differences between groups are reported as follows: °different from SNI;

§ different from FNO-SS;

# *different from FOE-SS*.

Comparison between clinical subgroups showed that disease duration was significantly different between them [One-way ANOVA F_(3, 838)_ = 12.17; *p* < 0.0001]. *Post hoc* analysis showed that disease duration was shorter in FNO-NS group than in all the other groups (*p* always < 0.001). No differences were found related to age (*p* > 0.05). Furthermore, prevalence of tremor elsewhere was different between groups (X^2^ = 323.00; *p* < 0.0001). Tremor elsewhere was less frequent in patients with FNO-NS than in the other groups (*p* always <0.0001).

### Associations Between Motor and Sensory Symptoms

Statistical analysis showed significant associations between tremor and sensory features in the various CD subtypes (Table 2).

**Table 2:** Associations between motor and sensory symptoms.

	**No Spread**		**Spread**	
	**FNO-NS**	**SNI**	**FNO-SS**	**FOE-SS**
HT-TE	**X**^**2**^ **=** **5.75;** ***p*** **=** **0.01**	**X**^**2**^ **=** **14.82;** ***p*** **<** **0.0001**	X^2^ = 4.07; *p* = 0.06	**X**^**2**^ **=** **7.02;** ***p*** **=** **0.010**
HT-ST	X^2^ = 0.36; *p* = 0.59	X^2^ = 2.41; *p* = 0.18	**X**^**2**^ **=** **4.99;** ***p*** **=** **0.04**	X^2^ = 0.16; *p* = 0.83
HT-pain	X^2^ = 2.53; *p* = 0.11	X^2^ = 0.08; *p* = 0.82	X^2^ = 0.25; *p* = 0.63	X^2^ = 0.45; *p* = 0.54
ST-TE	X^2^ = 1.58; *p* = 0.20	X^2^ = 0.002; *p* = 0.96	X^2^ = 0.04; *p* = 1	X^2^ = 0.43; *p* = 0.62
ST-pain	**X**^**2**^ **=** **28.09;** ***p*** **<** **0.0001**	X^2^ = 2.95; *p* = 0.12	X^2^ = 1.35; *p* = 0.31	X^2^ = 0.24; *p* = 0.67
pain-TE	X^2^ = 0.09; *p* = 0.85	X^2^ = 1.46; *p* = 0.31	X^2^ = 0.20; *p* = 0.79	X^2^ = 1.53; *p* = 0.24

*FNO-NS, focal neck onset, no spread; SNI, segmental neck involvement; FNO-SS, focal neck onset, segmental spread; FOE-SS, focal onset elsewhere, segmental spread to neck; HT, head tremor; TE, tremor elsewhere; ST, sensory trick. Bold is used for more relevant data*.

We found that in the CD patients who had focal neck onset without spread (FNO-NS; *p* = 0.016), in patients with segmental neck involvement without spread (SNI; *p* < 0.0001) and in patients with focal onset elsewhere with segmental spread to neck (FOE-SS; *p* = 0.010) head tremor was significantly associated with the presence of tremor elsewhere.

Furthermore, only in patients with focal neck onset and segmental spread, the presence of head tremor was significantly associated with the presence of sensory trick (FNO-SS; *p* = 0.040).

Finally, the presence of sensory trick was significantly associated with pain only in patients with focal neck onset, without spread (FNO-NS; *p* < 0.0001). Statistical analysis did not show any other significant associations.

### Logistic Regression Analysis

Results from logistic regression analysis of significant associations are reported in Table 3. Association between the presence of head tremor and tremor elsewhere in FNO-NS, SNI, and FOE-SS groups remained significant when adjusting for disease duration, sex and age. In addition, logistic regression analysis showed also a significant association between the presence of head tremor and disease duration (OR = 1.03, CI = 1.01–1.04, *p* < 0.0001) and sex (OR = 1.71, CI = 1.21–2.43, *p* < 0.002) in patients with FNO-NS, but not in patients in the other groups.

**Table 3:** Logistic regression analysis.

**Head Tremor (HT)**			
*FNO-NS*			
TE	OR = 2.63	CI: 1.18–5.85	***p* = 0.01**
adj. disease duration	OR = 2.61	CI: 1.16–5.85	***p* = 0.02**
adj. disease duration, sex	OR = 2.68	CI: 1.19–6.04	***p* = 0.01**
adj disease duration, sex, age	OR = 2.80	CI: 1.23–6.34	***p* = 0.01**
*SNI*			
TE	OR = 7.87	CI: 2.53–24.44	***p* < 0.0001**
adj. disease duration	OR = 8.10	CI: 2.58–25.36	***p* < 0.0001**
adj. disease duration, sex	OR = 7.78	CI: 2.41–25.04	***p* = 0.001**
adj disease duration, sex, age	OR = 8.05	CI: 2.48–26.04	***p* < 0.0001**
*FOE-SS*			
TE	OR = 4.61	CI: 1.54–13.76	***p* = 0.006**
adj. disease duration	OR = 4.46	CI: 1.48–13.41	***p* = 0.008**
adj. disease duration, sex	OR = 4.50	CI: 1.49–13.59	***p* = 0.008**
adj. disease duration, sex, age	OR = 5.45	CI: 1.71–17.33	***p* = 0.004**
**Sensory trick (ST)**			
*FNO-NS*			
Pain	OR = 2.75	CI: 1.88–4.02	***p* < 0.0001**
adj. disease duration	OR = 2.74	CI: 1.87–4.01	***p* < 0.0001**
adj. disease duration, sex	OR = 2.74	CI: 1.87–4.01	***p* < 0.0001**
adj disease duration, sex, age	OR = 2.67	CI: 1.82–3.92	***p* < 0.0001**
*FNO-SS*			
HT	OR = 3.71	CI: 1.25–10.99	***p* = 0.01**
adj. disease duration	OR = 3.74	CI: 1.26–11.13	***p* = 0.01**
adj. disease duration, sex	OR = 3.80	CI: 1.23–11.67	***p* = 0.02**
adj disease duration, sex, age	OR = 3.77	CI: 1.19–11.93	***p* = 0.02**

*FNO-NS, focal neck onset, no spread; SNI, segmental neck involvement; FNO-SS, focal neck onset, segmental spread; FOE-SS, focal onset elsewhere, segmental spread to neck; HT, head tremor; TE, tremor elsewhere; OR, odds ratio; CI, confidence interval. Bold is used for more relevant data*.

Finally, the association between sensory trick and neck pain described for patients in the FNO-NS group remained significant when adjusting for disease duration, sex and age. In addition, logistic regression analysis showed also a significant association between the presence of sensory trick and age (OR = 0.97, CI = 0.96–0.98, *p* < 0.0001) in patients with FNO-NS suggesting that younger age was associated with the use of sensory trick.

## Discussion

The main finding of the present study is that we found specific associations between motor (head tremor and tremor elsewhere) and sensory (sensory trick and pain) features in CD subgroups differing for the site of onset of dystonic symptoms and spread.

First, in several clinical subtypes of CD (FNO-NS, SNI, and FOE-SS) head tremor was significantly associated with the presence of tremor in body parts different from the head. Second, sensory trick was specifically associated with head tremor in focal neck onset with segmental spread and with pain in focal neck onset with no spread. These findings support the idea that these subgroups may represent distinct pathophysiological entities.

In our study following Norris et al. (12), CD patients were classified according to onset site of dystonia and spread. In our sample 70% of participants were classified as FNO-NS, 10% as SNI, 8% as FNO-SS and 12% as FOE-SS. Although prevalence of SNI and FOE-SS groups were similar between our study and the one reported by Norris et al. (12), the prevalence of FNO-NS and FNO-SS patients was slightly different. One possible explanation for the observed differences in prevalence of clinical subtypes may rely on the characteristics of spread assessment used in the two studies. The addition of quantitative measures in assessing dystonia in different body segments may be instrumental for making a more accurate diagnosis. In this regards, technological innovations, like video-analysis or the use of wearable sensors may be exploited for a quantitative analysis of dystonia (16, 17).

In our sample, patients with focal neck onset with no spread showed shorter disease duration compared to the other subgroups, suggesting that in patients with CD, spread could appear later during the course of dystonia. This observation is in accordance with the finding that in patients whose disease began with CD, focal dystonia could spread later, within 15 years from onset (18). Furthermore, tremor elsewhere was less frequent in the FNO-NS patients than in the other groups, in line with other studies where patients with segmental or multifocal dystonia were likely tremulous than patients with focal dystonia (12, 13, 19).

### Sensorimotor Subtypes

By analyzing sensorimotor associations in CD subgroups, classified on the basis of site of onset of dystonic symptoms and spread, we found a novel and interesting finding: in the majority of CD subgroups (FNO-NS, SNI, FOE-SS) the presence of head tremor was associated with the presence of tremor elsewhere. The association between head tremor and tremor elsewhere may suggest a common pathophysiological mechanism; i.e., an abnormal central oscillator in the cortico-subcortical network underpinning the expression of tremor. A possible candidate is the cerebellum. Recently Merola et al. suggested that tremor-dominant CD may be in the spectrum of the emerging “dystonia plus ataxia” syndrome, with patients with CD and tremor showing more severe ataxia and milder dystonia respect to patients without tremor (20). As already mentioned in the introduction, this clinical finding goes along with experimental (21) and neurophysiological (8–11) studies showing a strong association between tremor and abnormal cerebellar function in dystonia (7, 22).

Only in patients with FNO-NS the presence of head tremor is also associated with disease duration and sex, making females and patients with longer disease duration more predisposed to develop tremor. This finding confirms those of recent studies showing that patients with focal dystonia who had tremor had also a longer duration of symptoms (23) and were predominantly females (20).

The lack of association between head tremor and tremor elsewhere in patients with FNO-SS may indicate that the phenomenology of tremor might have been confounded with that of jerky dystonic contractions. A better characterization of “tremor” in dystonia may be helpful in distinguishing real oscillatory activity, associated with an abnormal central oscillator, from less regular jerky activity that may indeed be part of dystonic contractions. Moreover, it is necessary to take into account that the low numbers of FNO-SS patients with HT and TE could have influenced the statistical results (Table 1). With a larger population, in future studies, we might be able to clarify better if the association between HT/TE is present in all subgroups. Additionally, our large registry-based population will also offer the option to characterize sensorimotor features in all the cohort of CD patients, comparing patients with and without tremor.

Considering the sensory trick, we found that: (i) in patients with FNO-NS, there is a significant association between sensory trick and pain and between the presence of sensory trick and age; (ii) in patients with FNO-SS, there is a significant association between sensory trick and head tremor. These results may suggest a different mechanism of action of sensory trick in different CD subtypes: a gating mechanism attempting to reduce pain and a sensorimotor mechanism attempting to control head tremor.

Indeed, in patients with FNO-NS, we can assume that sensory trick is used in the attempt of pain modulation according to the “gate control theory” thanks to increased afferent feedback (24). Noteworthy, we also observed a significant association between the presence of sensory trick and age in patients with FNO-NS. The OR was minor than 1 suggesting that younger age was associated with sensory trick. Although age is not a mediator of the relationship between sensory trick and pain, this result suggests that younger patients are more likely to adopt sensory tricks, to possibly influence central processing of sensory inputs that contribute in reducing pain. However, this hypothesis related to the use of sensory tricks as an attempt to mitigate pain in FNO-NS, should be sustained with an appropriate study which, to the extent of our knowledge, it has not been carried out yet.

In patients with FNO-SS, the significant association between sensory trick and head tremor may suggest that sensory trick is used as “sensorimotor trick” to control the abnormal oscillations of the head. Unfortunately, we did not collect data regarding tremor and dystonia severity and its possible link with sensory trick. Thus, sensory trick may represent a sensorimotor mechanism aimed at compensating for the prolonged muscle spasm that characterizes cervical dystonia, tremor, or both. Despite these considerations, our results suggest that under the “umbrella” term sensory trick, clinicians likely include different maneuvers attempting to control for sensory (6, 25) or motor (26) aspects of the dystonic picture. A more accurate investigation of this intriguing aspect of dystonia is needed by developing an *ad-hoc* clinical instrument. Finally, it is worthy to underline that data on sensory trick were collected based on a yes/no answer and do not refer to the presence of a trick in the past. The present paper analyzed only baseline data collected in the IDR, however, since this registry includes an annual follow up for each patient, it is recommended in future studies to address the natural history of sensory trick and to include in such analysis the presence or omission of sensory trick in the past.

## Limitations

Some limitations of the study must be acknowledged.

Firstly, the present study is a retrospective registry-based study suffering of limitations due to its design: the registry does not include rating of severity of dystonia or tremor by appropriate scales or questionnaires, information about quality (i.e., tension-type, pulling-type, stretching, etc.) or intensity of pain. Furthermore, considering the high number of subjects enrolled in a registry, the dataset does not include objective measurements of motor or sensory features that can be obtained by means of neurophysiological techniques such as electromyography, kinematic analysis and evoked potentials recordings. We are aware that the lack of this information generates issues and interrogatives yet to be closed. For example it is not possible to address whether sensory trick is associated with a specific tremor phenotype in the FNO-SS group. A recent study supports the existence of two different sub-phenotypes of oscillatory head movements in CD, one characterized by large amplitude and jerky irregular pattern, the other characterized by small amplitude with a more regular and sinusoidal pattern (27). In addition we do not have information on whether the use of sensory trick is beneficial in reducing the intensity of pain or whether it is associated with a specific quality of pain. We acknowledge this limitation of the study, but at the same time we are confident that the results of the present study may be a useful tool to guide future researches on these specific topics.

Secondly, CD subtypes were classified using the site of onset and spread following recent observations (12), but across this profiling of CD subtypes we have to take into account that some subgroups are smaller (i.e., HT-TE in FNO-SS) compared to the others, leading to some statistical power issues. By the analysis of larger populations, this limitation, could be better defined in the future.

## Conclusions

In the present study we showed that different subgroups of CD clinically differing for site of onset and spread have different sensorimotor features. Even if this classification of CD subtypes is not supported by a strong neurobiological background, the clinical piece of information that emerged from the present study suggests the possibility that diverse pathophysiological mechanisms underlie the clinical variability of dystonic phenotypes. Further, our results highlight the necessity to define the characteristics of a quantitative assessment of dystonia to correctly classify CD subgroups, in order to obtain pathophysiological data and develop tailored pharmacological and non-pharmacological approaches.

## Data Availability Statement

The datasets generated for this study are available on request to the corresponding author.

## Ethics Statement

The studies involving human participants were reviewed and approved by Ethical Committee of Regione Liguria. The patients/participants provided their written informed consent to participate in this study.

## Author Contributions

FD, RM, GA, LA, and GDef: conceived and designed the experiments. FD, RM, GA, OB, LA, ME, FS, GT, AB, GFe, GFa, RP, REl, GDef, FB, DS, LB, MAl, VM, PB, REr, AA, CS, RL, MSC, GC, RC, MCM, MZ, AP, MP, MT, LMad, PG, LMag, SM, MR, BM, NM, MAg, DC, and GDev: performed the experiments. FD, RM, and LA: analyzed the data. FB, RM, GA, LA, AB, AA, and GDef: interpreted the data. FD, RM, GA, and LA: wrote the paper and drafted the article. FD, RM, GA, LA, AB, AA, and GDef: critically revised the article for important intellectual content. All authors contributed to the article and approved the submitted version.

## Conflict of Interest

The authors declare that the research was conducted in the absence of any commercial or financial relationships that could be construed as a potential conflict of interest.
